# Timely Access to Essential Surgery, Surgical Workforce, and Surgical Volume: Global Surgery Indicators in Mexico

**DOI:** 10.9745/GHSP-D-21-00745

**Published:** 2023-02-28

**Authors:** Rafael H. Pérez-Soto, Alicia Maybi Trolle-Silva, Gabriela Alejandra Buerba-Romero Valdés, Germán Esteban Sánchez-Morales, David Velázquez-Fernández, Antonio Ramos-De la Medina, Miguel F. Herrera

**Affiliations:** a Endocrine and Advanced Laparoscopic Surgery Service, Department of Surgery, National Institute of Medical Sciences and Nutrition Salvador Zubirán, Mexico City, Mexico.; b Department of Anatomical Pathology, National Institute of Medical Sciences and Nutrition Salvador Zubirán, Mexico City, Mexico.; c General Surgery Service, Department of Surgery, National Institute of Medical Sciences and Nutrition Salvador Zubirán, Mexico City, Mexico.; d Department of Surgery, Hospital Español de Veracruz, Veracruz, Mexico.

## Abstract

**Introduction:**

The Lancet Commission on Global Surgery seeks to improve surgical care outcomes and equity for the world population through 6 indicators outlined in its 2030 Global Surgery Report. Our study aimed to estimate the percentage of the Mexican population with access to surgical care within the 2-hour distance range (indicator 1), the surgical workforce density (indicator 2), and the number of surgical procedures performed per 100,000 inhabitants (indicator 3) during the year 2020. Knowing these indicators can help to design and implement policies to increase surgical care access coverage and equity in our country.

**Methods:**

Data related to population distribution, local referral hospitals, and surgical volume were obtained from the 2020 Mexican National Census. Information relating to hospital characteristics and surgical specialists was collected from the Secretariat of Health’s public records. We calculated travel time between health care facilities and municipalities using the TrueWay Matrix API and R Studio.

**Results:**

Taking into consideration the health care system affiliation, the proportion of the Mexican population with timely access to essential surgery was 81.7%, with 29.3 specialists per 100,000 inhabitants and 726.9 annual procedures performed per 100,000 inhabitants. We identified clusters of municipalities where a low proportion of the population has timely access to essential surgery.

**Conclusion:**

These findings illustrate that changes in Mexican policy are required to facilitate more equitable and timely access to essential surgical care among the population.

## Abstract (precis)

Our analysis of the 2020 national census data shows that while the majority of the Mexican population has timely access to essential surgery services, policy changes are needed to facilitate more equitable access to these services across the entire population.

## Teaser

**Key Findings:**

- In addition to geographical access to surgical care, other factors, including surgical workforce and surgical volume, affect access to essential surgical services.
- When considering Mexico’s fragmented public health system, an estimated 81.7% of the population have timely access to surgical care, but municipalities where population access was less than 20% were scattered throughout the country.
- Surgical workforce increase has been proportional to the Mexican population growth during the last 5 years, indicating no improvement in this area.

**Key Implication:**

- Policy changes are necessary to improve equitable and timely access to essential surgery services across the Mexican population.

## INTRODUCTION

Nearly 5 billion people worldwide lack access to safe and affordable surgical and anesthetic care, with those in low- and middle-income countries most impacted. In 2015, the Lancet Commission on Global Surgery (LCOGS) published a report describing the current state of global surgical care and providing recommendations for improving access and equity, with the goal of working toward universal health coverage. It also defined 6 national indicators to assess surgical care access, quality, and economic impacts on health systems (Table 1), accompanied by recommended goals for these indicators by 2030.^1^^–^^5^

**TABLE 1.** Lancet Commission on Global Surgery 2030 Indicators and Goals^5^

Indicator	Description	2030 Goals
Access to timely essential surgery	Proportion of the population that can access, within 2 hours, a facility that can do cesarean delivery, laparotomy, and treatment of open fracture	At least 80% coverage per country
Specialist surgical workforce density	Number of specialist surgical, anesthetic, and obstetric physicians who are working, per 100,000 population	100% of countries with at least 20 surgical, anesthetic, and obstetric physicians per 100,000 population
Surgical volume	Procedures done in an operating theater, per 100,000 population per year	100% of countries with a minimum 5,000 procedures per 100,000 population
Perioperative mortality	All-cause death rate before discharge in patients who have undergone a procedure in an operating theater, divided by the total number of procedures, presented as a percentage	100% countries tracking perioperative mortality
Protection against impoverishing expenditure	Proportion of households protected against impoverishment from direct out-of-pocket payments for surgical and anesthesia care	100% protection against impoverishment from out-of-pocket payments for surgical and anesthesia care
Protection against catastrophic expenditure	Proportion of households protected against catastrophic expenditure from direct out-of-pocket payment for surgical and anesthesia care	100% protection against catastrophic expenditure from out-of-pocket payments for surgical and anesthesia care

Several studies have focused their analysis on the need for and quality of surgical care, focusing on surgical volume; surgical, anesthesiology, and obstetric workforce; and perioperative morbidity/mortality. Nevertheless, the concept of access to surgical care remains poorly defined.^6^^–^^10^ Geographical information systems are valuable tools to assess health care access within a geographic region. Recent studies have successfully applied these tools to address the disparities in health care access throughout Africa.^11^^,^^12^ According to a study by Holmer et al. on global surgery indicators, only 19 countries have evaluated LCOGS Indicator 1, with only 2 of them from Latin America.^13^ Hanna et al. recently published a comprehensive situational analysis of the Colombian surgical system using the 6 core indicators.^14^ Indicators 1, 2, and 3 have never been defined for Mexico. Determining the geographical access to surgery, surgical workforce, and surgical volume at a national level will allow policymakers to develop programs that address disparities in surgical health care.

Determining the geographical access to surgery, surgical workforce, and surgical volume at a national level will allow policymakers to develop programs that address disparities in surgical health care.

According to the 2020 World Bank Country Classification, Mexico has an upper-middle-income economy.^15^ For a Mexican patient, public health care access, including surgical procedures, depends on the type of system with which the patient is affiliated. This means that not all health facilities or hospitals in Mexico are available for a particular individual. The Mexican public health care system is highly fragmented, and affiliation depends on employment status and employment sector (private or public) (Box). Approximately 23% of the Mexican population has private health care insurance.^16^

BOX **Health Care Subsystems in Mexico**

Instituto Mexicano del Seguro Social (Mexican Institute of Social Security) serves individuals with formal, nongovernment-related employment and their relatives (39.1%)

Instituto de Seguridad y Servicios Sociales de los Trabajadores del Estado (Institute of Security and Social Services for the State Workers) serves employees of the federal Mexican government and their families (7.7%)

Secretaría de Salud (Secretariat of Health) serves members of the population not affiliated with a system (49.9%)

Additional health systems exist for the Army (Secretaría de la Defensa Nacional) and Navy (Secretaría de Marina) and employees of the oil industry (Petróleos Mexicanos)

Our study aimed to assess what percentage of the Mexican population had access to essential surgical care (defined by the Bellwether procedures: urgent laparotomy, cesarean delivery, and open fracture treatment) within 2 hours (LCOGS Indicator 1). The secondary objective was to estimate the surgical workforce density (number of surgeons, anesthesiologists, and obstetricians per 100,000 people) (LCOGS Indicator 2), as well as the surgical volume per 100,000 people (LCOGS Indicator 3). These 3 indicators were estimated for the year 2020.

## METHODS

### Geographic and Population Data

We obtained Mexican population distribution data from the Secretariat of Welfare and the 2015 National Census of the National Institute of Statistics and Geography.^17^ Mexico comprises 32 states divided into municipalities that are further divided into localities (which are territorial and administrative divisions, each with a unique population nucleus and identity).^18^ Medical health insurance information, which determines the hospital networks/facilities that are available to individuals, was extracted from the 2020 National Census.^19^

### Health Facilities

We accessed updated information regarding national public health facilities and their catchment area/population from the Secretariat of Health website.^20^ Our analysis included active public health facilities that were affiliated with any public health insurance system and had surgical capabilities. This included facilities that were not traditional hospitals but harbored surgical capacity.

### Travel Time Calculations

For the purpose of travel time calculations, an algorithm was developed in RStudio version 1.3.959 for MacOS using the R programming language. This algorithm used the TrueWay Matrix Application Programming Interface (Rapid API) that provides travel duration (in seconds) and distance (in meters) for a set of origins and destinations. Origins and destinations were established based on geographic coordinates of each locality and the closest applicable health care facility, given their known health care subsystem and insurance network.

Appropriate, timely access to surgical care (LCOGS Indicator 1) was considered available if the minimum travel time for that population to an appropriate health facility with specific insurance coverage was less than 7,200 seconds (2 hours). The proportion of the population with timely access to surgical health care was calculated for the country, state, and municipality.

In scenarios where no health facilities were available in a specific municipality, travel times were calculated to the nearest facility in a municipality of the same state.

### Surgical Workforce Density Calculation

LCOGS Indicator 2 was calculated using the total number of surgical, orthopedic, anesthesiology, and obstetric physicians per 100,000 population of each of the 32 states.^21^

### Surgical Procedure Rate Calculation

We estimated LCOGS Indicator 3 using the total number of surgical procedures performed in public health facilities and the total population of each of the 32 states. Data were once again accessed from the public Secretariat of Health records. Although data from the public health system were available, the COVID-19 pandemic significantly reduced the number of surgical procedures performed in 2020, making this indicator unreliable and not properly representative of the public health system’s capacity to maintain surgical volume.

### Map Data Construction

We constructed geographic data maps using QGIS Bucuresti version 3.12.3. Vectorial layers for Mexico’s geography, including political and territorial geographic distribution by states, municipalities, and localities, were downloaded from the Comisión Nacional para el Conocimiento y Uso de la Biodiversidad (CONABIO) website and used for map layer elaboration.^22^

### Ethical Approval

We obtained approval from the National Institute of Medical Sciences and Nutrition Salvador Zubirán Institutional Review Board (CIBH 3644).

## RESULTS

**FIGURE 1:**
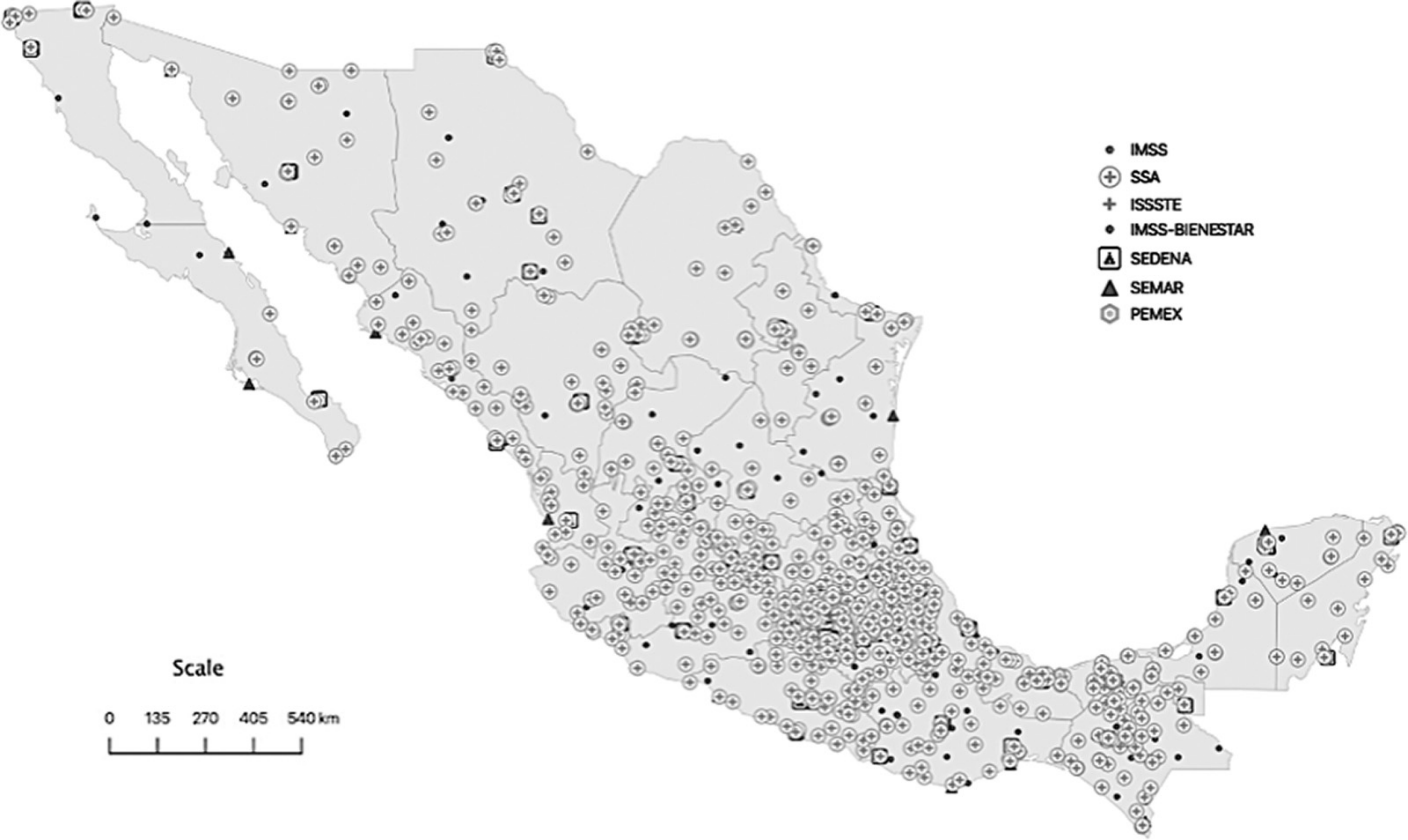
Geographic Distribution of Health Facilities in Mexico in 2020 Abbreviations: IMSS, Instituto Mexicano del Seguro Social; ISSSTE, Instituto de Seguridad y Servicios Sociales de los Trabajadores del Estado; PEMEX, Petróleos Mexicanos; SEDENA, Secretaría de la Defensa Nacional; SEMAR, Secretaría de Marina; SSA, Secretaría de Salud.

In 2020, there were 1,594 health facilities with Bellwether procedure capabilities distributed throughout the country. These were located in 719 (29.1%) of the 2,469 municipalities and 804 (0.2%) of the 300,690 localities. Of the 1,594 facilities, 933 (58.6%) were affiliated with Secretaría de Salud, 300 (18.8%) with Instituto Mexicano del Seguro Social (IMSS), 123 (7.7%) with Instituto de Seguridad y Servicios Sociales de los Trabajadores del Estado, 96 (6.0%) with the Secretaría de la Defensa Nacional, 83 (5.2%) with IMSS-Bienestar, 35 (2.2%) with Secretaría de Marina, and the remaining 24 (1.5%) with the Petróleos Mexicanos health system. Figure 1 displays the geographic distribution of health facilities in Mexico in 2020.

### Access to Timely Essential Surgery

**FIGURE 2:**
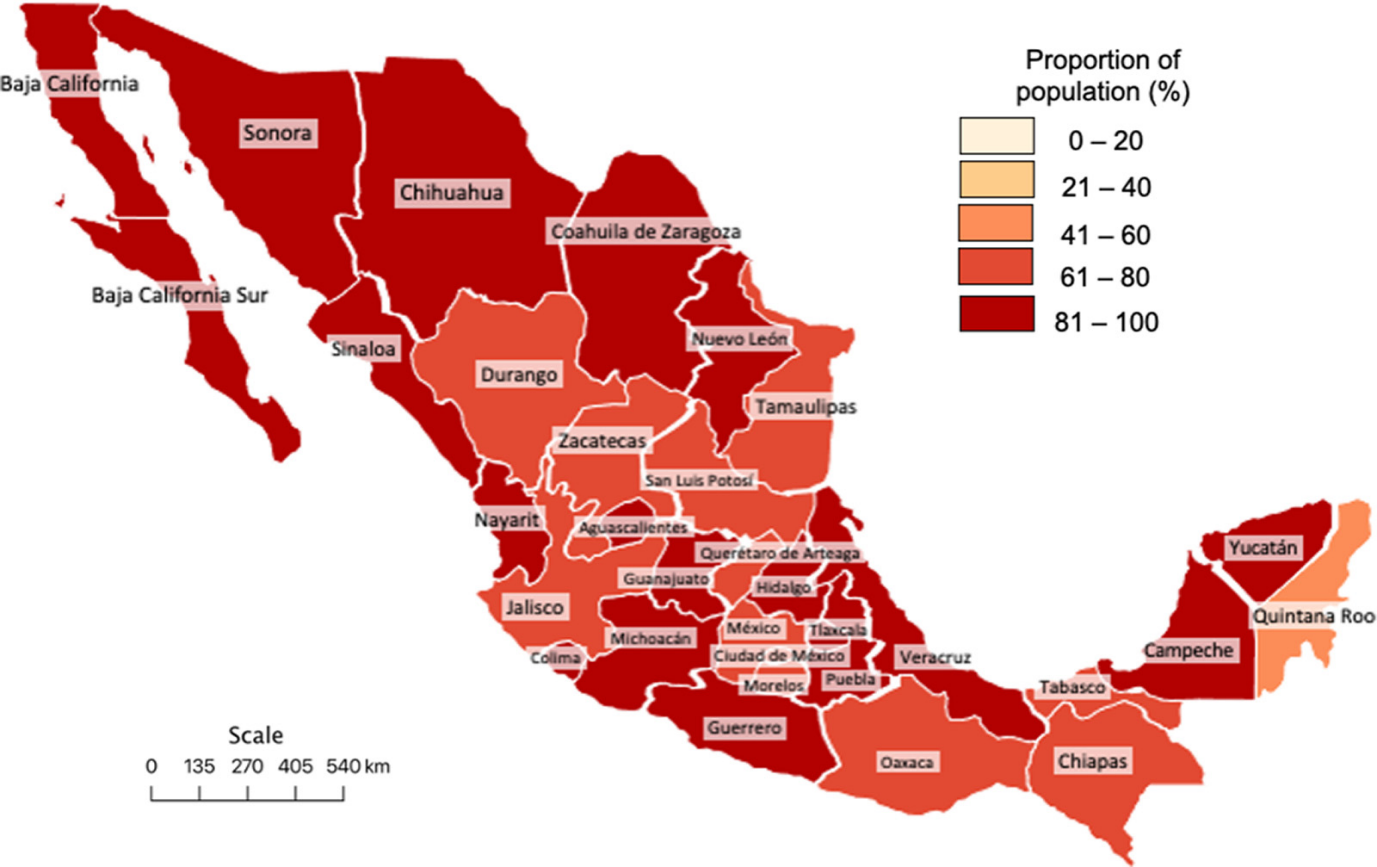
Proportion of Population With Timely Access to Surgical Care Among Mexican States in 2020

**FIGURE 3:**
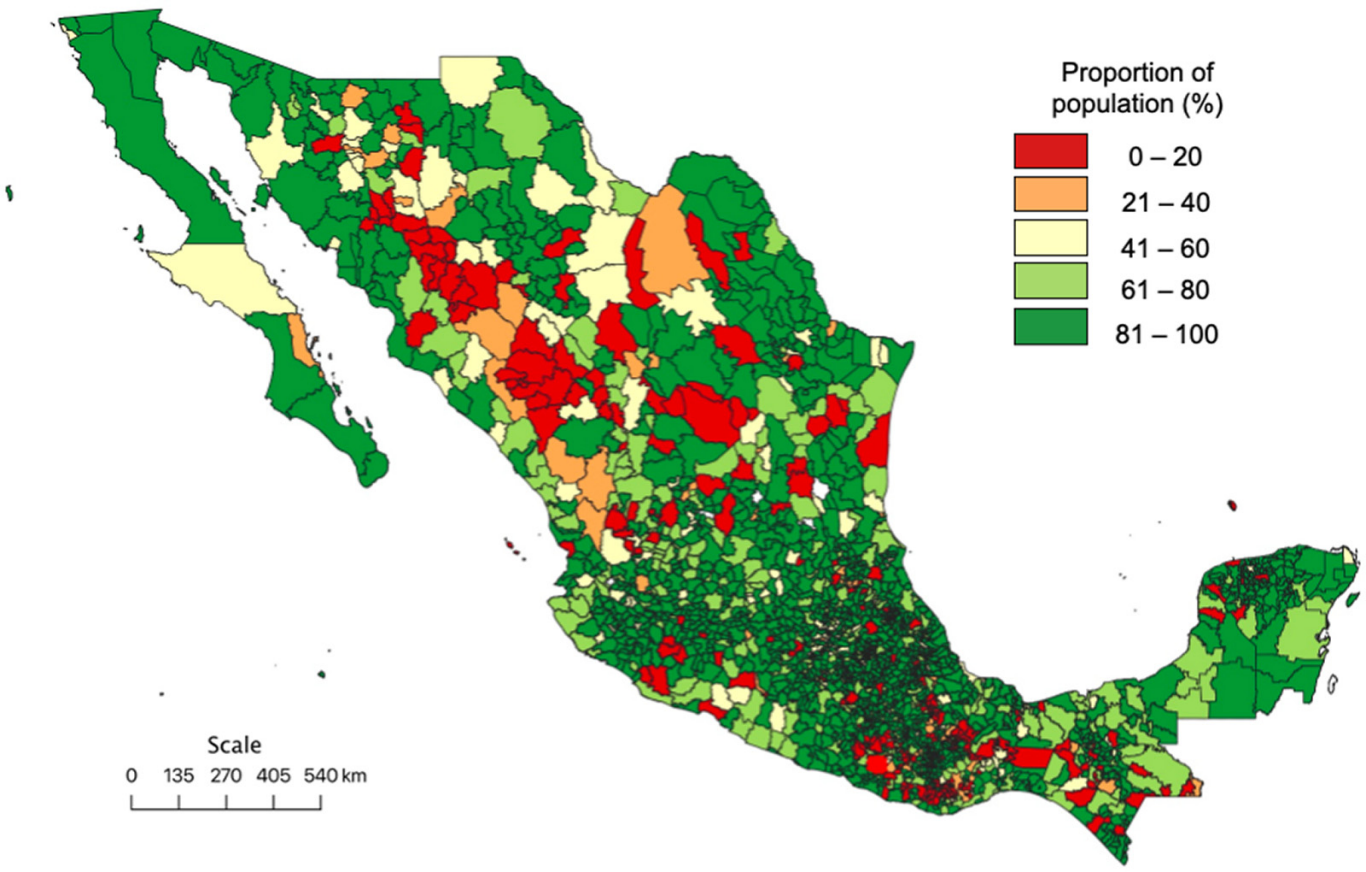
Proportion of Population With Timely Access to Surgical Care Among Mexican Municipalities in 2020

A total of 1,393,479 travel time calculations were performed between localities and health facilities. Data from 13,962 calculations were not available, representing 1% of the travel time queries. Based on localities alone, 96.1% of the Mexican population had timely (<2 hour) access to a facility that could perform Bellwether procedures. However, in a more appropriate analysis, when distances were calculated based on the nearest facility appropriate for an individual’s health insurance, the proportion of the population with timely access to surgical care was 81.7%. The average estimate for LCOGS Indicator 1 stratified by state was 81.2% (range: 53.3%–94.2%). Individual data for each of the 32 states are shown in Table 2. Choropleth maps representing the population proportion with timely access to surgical care in 2020 among the individual states and municipalities are shown in Figures 2 and 3, respectively.

**TABLE 2.** Demographic Data, Access to Timely Essential Surgery, Surgical Workforce Density, and Surgical Volume for the 32 Mexican States in 2020

State	Population2020, No.	Localities,No.	COVID-19Cases per100,000Persons	Population WithAccess to SurgicalCare <2 Hours,No. (%)	SurgicalSpecialistWorkforce, No.(SurgicalSpecialist per100,000Inhabitants)	SurgicalProcedures,No.	SurgicalProceduresper 100,000Persons, No.
Northwest							
Baja California	3,769,020	5,545	993.20	3,383,533 (89.77)^a^	4,097 (108.70)	15,751	417.90
Baja California Sur	798,447	2,543	2,275.41	735,181 (92.07)^a^	1,598 (200.13)	7,450	933.06
Chihuahua	3,741,869	12,186	1,065.40	3,182,528 (85.05)^a^	4,897 (130.87)	23,978	640.80
Durango	1,832,650	5,890	1,439.99	1,196,336 (65.27)	2,751 (150.11)	16,481	899.29
Sinaloa	3,026,943	5,495	913.85	2,683,966 (88.66)^a^	4,691 (154.97)	18,981	627.06
Sonora	2,944,840	7,300	1,925.40	2,717,896 (92.29)^a^	4,981 (169.14)	21,556	731.99
Northeast							
Coahuila de Zaragoza	3,146,771	4,034	1,684.42	2,770,363 (88.03)^a^	4,599 (146.14)	11,729	372.73
Nuevo León	5,784,442	4,822	1,479.15	4,692,210 (81.11)^a^	6,402 (110.67)	20,808	359.72
Tamaulipas	3,527,735	6,566	1,195.27	2,817,000 (79.85)	5,562 (157.66)	24,309	689.08
West							
Colima	731,391	1,126	1,097.22	689,356 94.25)^a^	1,459 (199.48)	6,225	851.11
Jalisco	8,348,151	10,348	658.25	6,379,923 (76.42)	9,927 (118.91	53,459	640.36
Michoacán de Ocampo	4,748,846	8,644	726.28	4,222,771 (88.92)^a^	5,626 (118.47)	33,231	699.77
Nayarit	1,235,456	2,850	648.82	1,078,681 (87.31)^a^	2,090 (169.16)	7,285	589.66
East							
Hidalgo	3,082,841	4,690	813.01	2,544,173 (82.52)^a^	3,911 (126.86)	24,688	800.81
Puebla	6,583,278	6,568	770.55	5,811,371 (88.27)^a^	7,271 (110.44)	44,481	675.66
Tlaxcala	1,342,977	1,175	883.33	1,131,686 (84.26)^a^	1,906 (141.92)	12,119	902.39
Veracruz de Ignacio de la Llave	8,062,579	19,845	556.45	6,717,601 (83.31)^a^	10,772 (133.60)	59,491	737.86
North Center							
Aguascalientes	1,425,607	2,022	1,262.54	1,285,601 (90.17)^a^	2,354 (165.12)	18,573	1302.81
Guanajuato	6,166,934	8,809	1,398.87	5,417,006 (87.83)^a^	7,649 (124.03)	92,217	1495.34
Querétaro	2,368,467	2,192	1,449.67	1,722,168 (72.71)	2,944 (124.29)	20,642	871.53
San Luis Potosí	2,822,255	6,554	1,492.81	2,277,295 (80.69)^a^	3,185 (112.85)	18,909	669.99
Zacatecas	1,622,138	4,498,	1,324.60	1,070,288 (65.98)	2,464 (151.89)	23,378	1441.18
South Center							
Ciudad de México	9,209,944	634	3,933.52	7,354,274 (79.85)	25,572 (277.65)	77,930	846.15
Estado de México	16,992,418	4,894	952.24	12,256,308 (72.12)	17,689 (104.09)	93,326	549.22
Morelos	1,971,520	1,578	580.11	1,337,400 (67.83)	2,814 (142.73)	13,719	695.85
Southwest							
Chiapas	5,543,828	21,157	153.55	4,310,336 (77.75)	5,969 (107.66)	29,501	532.14
Guerrero	3,540,685	6,769	754.57	3,057,466 (86.35)^a^	5,938 (167.70)	32,290	911.97
Oaxaca	4,132,148	10,723	719.67	2,973,945 (71.97)	5,333 (129.06)	26,082	631.19
Southeast							
Campeche	928,363	2,762	811.75	761,749 (82.05)^a^	1,879 (202.39)	7,117	766.61
Quintana Roo	1,857,985	2,207	867.33	991,689 (53.37)	2,341 (125.99)	13,138	707.11
Tabasco	2,402,598	2,472	1,861.77	1,898,068 (79.00)	4,639 (193.08)	28,766	1197.28
Yucatán	2,320,898	2,434	1,156.10	1,973,427 (85.02)^a^	3,752 (161.66)	18,150	782.02
Total	126,014,024	189,432	1,208.49	102,967,672 (81.71)^a^	36,973 (29.34)	916,120	726.99

a Goal indicator accomplished.

### Surgical Workforce Density

The most recent data for the surgical specialist workforce are from 2019. According to the Secretariat of Health, there were 10,823 obstetricians, 13,111 surgical specialists (9,078 surgeons and 4,033 orthopedists), and 13,039 anesthesiologists in the public health system. Using the total Mexican population from the 2020 census (126,014,024 inhabitants), LCOGS Indicator 2 estimates suggest that there were 8.6 obstetricians per 100,000 inhabitants, 10.4 surgical specialists per 100,000 inhabitants, and 10.3 anesthesiologists per 100,000 inhabitants.

### Surgical Volume

Surgical volume for 2020 was 916,120 procedures, suggesting LCOGS Indicator 3 was approximately 726.9 procedures per 100,000 inhabitants.

## DISCUSSION

Based on our initial calculation, 96.1% of the Mexican population had timely access to a health facility with the capacity for surgical care based on geographic location alone. Although these data appear to reflect a strong indicator, the Mexican health system’s accessibility is fragmented by insurance programs. When considering these systems, the estimate drops to 81.7%, a more accurate representation of the country’s accessibility status.

Recently, Carrillo-Villaseñor et al. estimated that 77.9% of the state population of Chiapas, Mexico had timely access to surgical care, only accounting for 24-hour surgical facilities.^23^ The proportion reported in their study is consistent with our estimation for this state (77.7%). In addition, these authors’ geographical distribution of the population with access to surgical care within 2 hours was congruent with our findings.

After comparing this indicator between Mexico and previously reported data from 3 other upper-middle-income Latin American countries (Belize, St. Vincent and Grenadines, and Brazil), we noticed inferior timely access to surgical care in the Mexican population. This can partially be explained due to a larger Mexican population—more than 120 million inhabitants, compared to 109,148 inhabitants in St. Vincent and Grenadines and 360,933 inhabitants in Belize—in addition to a more vast national territory.^24^ Furthermore, Belize has only 1 public health care system, in contrast with Mexico’s fragmented public health infrastructure comprising at least 6 different health care subsystems.^25^ Brazil’s estimation of LCOGS Indicator 1 (97.2%) as reported by Massenburg et al. highlights the potential impact of a unified national health system.^26^

Municipalities with the lowest proportion of individuals with timely access to surgical care are distributed throughout Mexico, with a tendency to form clusters (Figure 3, red highlight). Although the primary aim of this study was to estimate the LCOGS Indicator 1 in Mexico, identification of these clusters is one of the most important findings, as these regions are susceptible to health care access improvement through the development of new public health policies.

Identification of clusters of municipalities with the lowest proportion of individuals with timely access to surgical care is one of the most important findings of our study.

Regarding the surgical specialist workforce, data showed an estimation for LCOGS Indicator 2 of 8.6 obstetricians per 100,000 inhabitants, 10.4 surgical specialists per 100,000 inhabitants, and 10.3 anesthesiologists per 100,000 inhabitants. Workforce increase has been proportional to the Mexican population growth during the last 5 years; therefore, no improvement has occurred in this area. According to our estimate for 2020, Mexico lacks health specialists, as the goal for this indicator is 20 specialists of each type (surgeon, obstetrician, and anesthesiologist) per 100,000 inhabitants.

It is difficult to make conclusions regarding the surgical volume for the year 2020, as the number of surgical procedures performed was significantly impacted by the COVID-19 pandemic. Countrywide health facilities were required to reallocate their human, financial, and medical equipment resources, detracting from other medical engagements. As reported before the COVID-19 pandemic by the Secretariat of Health, national surgical volume was 1,462,910 procedures in 2017 (1,172.2 procedures per 100,000 inhabitants), 1,361,779 in 2018 (1,079.1 procedures per 100,000 inhabitants), and 1,308,578 in 2019 (1,025.5 procedures per 100,000 inhabitants). Data from COVID-19 cases in 2020 by state is displayed in Table 2.^27^

Our estimation for this indicator was 726.9 procedures per 100,000 inhabitants, which is a significantly inferior surgical volume when compared to the years prior. In 2019, Esqueda-Nuñez, et al. described the volume of surgical procedures in Mexico for the year 2015 stratified by states.^28^ According to their findings, 3 states (Jalisco, Baja California Sur, and Nuevo León) had more than 5,000 surgical procedures per 100,000 inhabitants. According to Holmer et al., estimated surgical volumes for Mexico approach the calculated median (interquartile range [IQR]) surgical volume in the Americas and upper-middle-income countries, estimated around 3,005 (IQR: 1,744–4,800) and 3,375 (IQR: 2,034–12,352), respectively.^16^ When compared with countries of Latin America, the Mexican surgical volume is below the averages for Brazil, Colombia, Cuba, Nicaragua, and Peru. Unfortunately, contrasting our results with previously published data is not appropriate given the impacts of the COVID-19 pandemic.

As suggested by Davies et al., access cannot be defined as just a matter of geographical proximity. In their review of the LCOGS goals, they address the importance of collecting detailed population data 1x1 km (including age and sex), facility location and surgical capability, and the type of facility (public vs. private facilities; primary, secondary, or tertiary centers).^29^ We share this proposed definition of access to surgery, as many factors influence access in a fragmented health care system such as that found in Mexico.

Unifying the public health system proposes an interesting solution to accomplish surgical care delivery for the entire Mexican population, given that it would increase the true access rate to 96.1%. Changing the functional mechanisms and policies of an entire resource-limited public health system would require considerable time for planning, implementation, and improvement. Meanwhile, temporal solutions may be employed, such as a national policy that allows patients to access any health facility if any urgent surgical, obstetric, or orthopedic treatment is needed.

We are aware that some modifications in the Mexican public health system have been implemented to improve its infrastructure, increasing health care accessibility. An example of this is the IMSS-Bienestar program, running for more than 40 years and designed to provide health care in rural or marginal urban regions (including surgical care) distributed in the entire country. Other programs have addressed different global surgery indicators, such as the training of rural surgeons and rural anesthesiologists, as well as the creation of new medical schools and residency programs, which have a direct impact on workforce indicators.^30^ Nevertheless, timely access to surgical care is currently an infrastructure problem; therefore, national programs directed to increase the health system infrastructure network are needed. The national surgical plan framework recommendations proposed by the LCOGS include exploring policies to equip first-level health facilities to provide surgical care and enhancing referral systems.^5^

Timely access to surgical care depends on many factors other than the presence of a nearby health facility. Lack of suitable transport, patient finances, lower literacy, lack of hospital bed space, and patient overload have been reported.^31^^–^^33^ This implies that increasing the health system and national road infrastructure are not the only solutions to achieve 100% surgical care coverage. Government investments in health human resources, medical equipment, public education, and social development programs are required on an ongoing basis. A future study addressing the interactions and impact of these factors on the access to high-quality surgical care in Mexico through neural network modeling is underway.

Timely access to surgical care depends on many factors other than proximity to a health facility.

### Limitations

Limitations of this study were mitigated but not entirely avoidable. First, data on low-volume population localities were summarized and not reported individually in the 2020 National Mexican Census. Second, our travel time calculations may underestimate the actual time required for travel and may overestimate access to surgical care facilities as optimistic parameters were used to search for these (toll usage and transportation by car). In addition, calculations for the year 2020 were performed in 2022, which may have led to a marginal change in travel time because of infrastructure developments that occurred in the interim. Moreover, information regarding 1% of the travel time calculations for a specific origin-destination binomial was not available and was not included in our assessment.

## CONCLUSION

While our estimates show that a majority of the Mexican population has timely access to essential surgical care, we identified clusters of municipalities where a low proportion of inhabitants have this access. According to our estimates, Mexico is also lacking in surgical specialists. Our data serve as a justification and starting point for the development of future public health policies to address inequities in access to essential surgical services in Mexico. This development pertains not only to the health care system directly but also to all aspects of government and infrastructure, such as the Ministry of Communications and Transportation, the Secretary of Economy, and other government agencies.

## Notes

**Peer Reviewed**

**First published online:** February 2, 2023.

## References

[1] Dare AJ Grimes CE Gillies R. Global surgery: defining an emerging global health field. Lancet. 2014;384(9961):2245–2247. 10.1016/S0140-6736(14)60237-3. 24853601

[2] Farmer PE Kim JY. Surgery and global health: a view from beyond the OR. World J Surg. 2008;32(4):533–536. 10.1007/s00268-008-9525-9. 18311574 PMC2267857

[3] Bath M Bashford T Fitzgerald JE. What is ‘global surgery’? Defining the multidisciplinary interface between surgery, anaesthesia and public health. BMJ Glob Health. 2019;4(5):e001808. 10.1136/bmjgh-2019-001808. 31749997 PMC6830053

[4] Ozgediz D Riviello R. The “other” neglected diseases in global public health: surgical conditions in sub-Saharan Africa. PLoS Med. 2008;(6):e121. 10.1371/journal.pmed.0050121. 18532875 PMC2408612

[5] Meara JG Leather AJM Hagander L. Global Surgery 2030: evidence and solutions for achieving health, welfare, and economic development. Lancet. 2015;386(9993):569–624. 10.1016/S0140-6736(15)60160-X. 25924834

[6] Holmer H Lantz A Kunjumen T. Global distribution of surgeons, anaesthesiologists, and obstetricians. Lancet Glob Health. 2015;3(Suppl 2):S9–S11. 10.1016/S2214-109X(14)70349-3. 25926323

[7] Holmer H Shrime MG Riesel JN Meara JG Hagander L. Towards closing the gap of the global surgeon, anaesthesiologist, and obstetrician workforce: thresholds and projections towards 2030. Lancet. 2015;385(Suppl 2):S40. 10.1016/S0140-6736(15)60835-2. 26313089

[8] Shrime MG Dare A Alkire BC Meara JG. A global country-level comparison of the financial burden of surgery. Br J Surg. 2016;103(11):1453–1461. 10.1002/bjs.10249. 27428044

[9] Anderson GA Ilcisin L Abesiga L. Surgical volume and postoperative mortality rate at a referral hospital in Western Uganda: Measuring the Lancet Commission on Global Surgery indicators in low-resource settings. Surgery. 2017;161(6):1710–1719. 10.1016/j.surg.2017.01.009. 28259351

[10] Rickard JL Ntakiyiruta G Chu KM. Associations with perioperative mortality rate at a major referral hospital in Rwanda. World J Surg. 2016;40(4):784–790. 10.1007/s00268-015-3308-x. 26546186

[11] Esquivel MM Uribe-Leitz T Makasa E. Mapping disparities in access to safe, timely, and essential surgical care in Zambia. JAMA Surg. 2016;151(11):1064–1069. 10.1001/jamasurg.2016.2303. 27580500 PMC5179136

[12] Juran S Broer PN Klug SJ. Geospatial mapping of access to timely essential surgery in sub-Saharan Africa. BMJ Glob Health. 2018;3(4):e000875. 10.1136/bmjgh-2018-000875. 30147944 PMC6104751

[13] Holmer H Bekele A Hagander L. Evaluating the collection, comparability and findings of six global surgery indicators. Br J Surg. 2019;106(2):e138–e150. 10.1002/bjs.11061. 30570764 PMC6790969

[14] Hanna JS Herrera-Almario GE Pinilla-Roncancio M. Use of the six core surgical indicators from the Lancet Commission on Global Surgery in Colombia: a situational analysis. Lancet Glob Health. 2020;8(5):e699–e710. 10.1016/S2214-109X(20)30090-5. 32353317

[15] World Bank country and lending groups. 2020 country classification. The World Bank. Accessed January 4, 2023. https://datahelpdesk.worldbank.org/knowledgebase/articles/906519-world-bank-country-and-lending-groups

[16] Datos en relación a salud y seguridad social (derecho habiencia). INEGI. Accessed January 4, 2023. https://www.inegi.org.mx/temas/derechohabiencia/

[17] Catálogo Único de Claves de Áreas Geoestadísticas Estatales, Municipales y Localidades. Programas de información. Instituto Nacional de Estadística y Geografía. Accessed January 16, 2023. https://www.inegi.org.mx/app/ageeml/

[18] Archivo histórico de localidades geoestadísticas, catálogos predefinidos. INEGI. Accessed January 4, 2023. https://www.inegi.org.mx/app/geo2/ahl/

[19] Sistema de Consulta/México en Cifras/publicaciones. INEGI. Accessed January 4, 2023. https://www.inegi.org.mx/app/areasgeograficas/?ag=00#collapse-Indicadores

[20] Catálogos CLUES. Secretaría de Salud. Dirección General de Información en Salud, Intercambio de información. Accessed January 4, 2023. http://www.dgis.salud.gob.mx/contenidos/intercambio/clues_gobmx.html

[21] DGIS, cubos dinámicos. Secretaría de Salud. Accessed January 4, 2023. http://www.dgis.salud.gob.mx/contenidos/basesdedatos/BD_Cubos_gobmx.html

[22] Geoportal del Sistema Nacional de Información sobre Biodiversidad – CONABIO. Accessed January 4, 2023. http://www.conabio.gob.mx/informacion/gis/

[23] Carrillo-Villaseñor F Fowler Z Moeller E. Access to essential surgical care in Chiapas, Mexico: a system-wide geospatial analysis. World J Surg. 2021;45(6):1663–1671. 10.1007/s00268-021-05975-y. 33616710

[24] World development indicators. The World Bank. Accessed January 4, 2023. http://datatopics.worldbank.org/world-development-indicators/

[25] Organización Panamericana de la Salud (OPS). Salud en las Américas, Edición de 2017. *Resumen: Panorama Regional y Perfiles del País*. OPS; 2017. Accessed January 4, 2023. https://iris.paho.org/handle/10665.2/34322

[26] Massenburg BB Saluja S Jenny HE. Assessing the Brazilian surgical system with six surgical indicators: a descriptive and modelling study. BMJ Glob Health. 2017;2(2):e000226. 10.1136/bmjgh-2016-000226. 28589025 PMC5444087

[27] Covid-19 México. Government of Mexico. Accessed January 4, 2023. https://datos.covid-19.conacyt.mx

[28] Esqueda-Nuñez R Durán V. Panorama estadístico de las intervenciones quirúrgicas en México de 2000 a 2017. Presented at: XLIII Congreso Internacional de Cirugía General 2019; October 13–18, 2019; León, Guanajuato, Mexico. 10.13140/RG.2.2.35200.79361

[29] Davies JI Gelb AW Gore-Booth J. Global surgery, obstetric, and anaesthesia indicator definitions and reporting: an Utstein consensus report. PLoS Med. 2021;18(8):e1003749. 10.1371/journal.pmed.1003749. 34415914 PMC8415575

[30] Gobierno de México. Instituto Mexicano del Seguro Social (IMSS). Libro Blanco. *Programa IMSS-Prospera 2013-2018*. IMSS; 2018. Accessed January 4, 2023. https://www.imss.gob.mx/sites/all/statics/pdf/transparencia/rendicion/2012-2018-LB-4-IMSS-PROSPERA.pdf

[31] Varela C Young S Mkandawire N Groen RS Banza L Viste A. Transportation barriers to access health care for surgical conditions in Malawi: a cross sectional nationwide household survey. BMC Public Health. 2019;19(1):264. 10.1186/s12889-019-6577-8. 30836995 PMC6402149

[32] Boeck MA Nagarajan N Gupta S. Assessing access to surgical care in Nepal via a cross-sectional, countrywide survey. Surgery. 2016;160(2):501–508. 10.1016/j.surg.2016.03.012. 27158120

[33] Nwanna-Nzewunwa OC Ajiko MM Kirya F. Barriers and facilitators of surgical care in rural Uganda: a mixed methods study. J Surg Res. 2016;204(1):242–250. 10.1016/j.jss.2016.04.051. 27451893

